# **k**-space imaging of anisotropic 2D electron gas in GaN/GaAlN high-electron-mobility transistor heterostructures

**DOI:** 10.1038/s41467-018-04354-x

**Published:** 2018-07-11

**Authors:** L. L. Lev, I. O. Maiboroda, M.-A. Husanu, E. S. Grichuk, N. K. Chumakov, I. S. Ezubchenko, I. A. Chernykh, X. Wang, B. Tobler, T. Schmitt, M. L. Zanaveskin, V. G. Valeyev, V. N. Strocov

**Affiliations:** 1 Swiss Light Source, Paul Scherrer Institute, 5232 Villigen-PSI, Switzerland; 2 National Research Centre “Kurchatov Institute”, 123182 Moscow, Russia; 3 National Institute of Materials Physics, Atomistilor 405A, 077125 Magurele, Romania

## Abstract

Nanostructures based on buried interfaces and heterostructures are at the heart of modern semiconductor electronics as well as future devices utilizing spintronics, multiferroics, topological effects, and other novel operational principles. Knowledge of electronic structure of these systems resolved in electron momentum k delivers unprecedented insights into their physics. Here we explore 2D electron gas formed in GaN/AlGaN high-electron-mobility transistor heterostructures with an ultrathin barrier layer, key elements in current high-frequency and high-power electronics. Its electronic structure is accessed with angle-resolved photoelectron spectroscopy whose probing depth is pushed to a few nanometers using soft-X-ray synchrotron radiation. The experiment yields direct **k**-space images of the electronic structure fundamentals of this system—the Fermi surface, band dispersions and occupancy, and the Fourier composition of wavefunctions encoded in the k-dependent photoemission intensity. We discover significant planar anisotropy of the electron Fermi surface and effective mass connected with relaxation of the interfacial atomic positions, which translates into nonlinear (high-field) transport properties of the GaN/AlGaN heterostructures as an anisotropy of the saturation drift velocity of the 2D electrons.

## Abstract (web summary)

Semiconductor heterostructures hosting two-dimensional electron gases are widely used today in high-electron-mobility transistors. Here, the authors probe the electronic structure in GaN/AlGaN, heterostructures, discovering planar anisotropy of the electron Fermi surface, offering new insights into transport properties.

## Introduction

The concept of high-electron-mobility transistors (HEMTs) advanced by Mimura^1^ in the early 80s for GaAs/GaAlAs heterostructures revolutionized the field of high-frequency semiconductor electronics. It exploits an idea of polarization engineering when a large band offset between an intrinsic semiconductor and a doped barrier layer forms a quantum well (QW) at the interface that confines a mobile two-dimensional electron gas (2DEG) on the intrinsic semiconductor side. Its spatial separation from defects in the doped barrier layer and in the interface region—in contrast to conventional transistor structures where the 2DEG is formed by doping—allows the electrons to escape defect scattering and dramatically increase their mobility $\mu _{\mathrm{e}}$, limited then only by phonon scattering. This fundamental operational principle of HEMTs boosts their high-frequency performance, which is exploited in a wide spectrum of applications such as cell phones.

A characteristic property of HEMTs based on wurtzite GaN/AlGaN heterostructures is the accumulation of large sheet carrier concentrations *n*_s_ ~ 10^13^ cm^−2^—about one order of magnitude higher compared with other III–V or elementary semiconductors^2,3^—without intentional doping of the barrier layer. This property is attributed to the formation of a deep spike-shaped QW at the heterojunction, where a large conduction band offset coexists with large piezoelectric and spontaneous polarization^4,5^. Although $\mu _{\mathrm{e}}$ in GaN-HEMTs is limited by a relatively large electron effective mass m* ~ 0.22 in bulk GaN, nearly three times larger than in GaAs, these devices demonstrate an advantageous combination of sufficiently high operating frequency with exceptionally high current density, resulting from the large *n*_s_, saturation drift velocity, operating temperature, and breakdown voltage. These advantages make the GaN-HEMTs indispensable components of high-power amplifiers for microwave communication and radar systems. Recently, the ideas of creating mobile 2DEGs using spontaneous and strain-induced polarization at the interface have been extended to oxide systems such as the binary Mg_*x*_Zn_1-*x*_O/ZnO,^6^ LaAlO_3_/SrTiO_3_^7^, and CaZrO_3_/SrTiO_3_^8^ heterostructures that typically embed orders of magnitude larger *n*_s_.

The state-of-art GaN-HEMTs operate nowadays at the edge of their physical limitations, which remain far from complete understanding. Development of new strategies to improve their performance and conquer the near-THz operational range^9^ needs qualitatively new experimental knowledge about the physics of these devices. Particularly important is **k**-space information about the Fermi surface (FS), band dispersions, and wavefunctions of the embedded 2DEG. These fundamental electronic structure characteristics, only indirectly accessible in optics and magnetotransport experiments such as the Hall effect, Shubnikov-de Haas oscillations, cyclotron resonance, etc.^10–12^, can be directly probed using the **k**-resolving technique of angle-resolved photoelectron spectroscopy (ARPES). However, the small photoelectron mean free path *λ*_PE_ in conventional ARPES with photon energies *hv* around 100 eV limits its depth sensitivity to ~ 0.5 nm. Access to buried electron systems such as the HEMTs requires pushing this technique to the soft-X-ray energy range (SX-ARPES, see a recent review ref. 13) with *hv* around 1 keV and higher, where *λ*_PE_ grows with photoelectron kinetic energy as ~ *E*_k_^3/4^ ^14,15^. For GaN in particular, elastic-peak electron spectroscopy measurements^16^ show that an increase of *E*_k_ from 200 eV to 2 keV results in an increase of *λ*_PE_ from ~ 0.5 to 4 nm. An added virtue of SX-ARPES, still largely overlooked in applications to 2D systems such as QW states (QWSs), is a significantly sharper definition of the out-of-plane component *K*_*z*_ of the final-state momentum **K**. This fact results from the larger *λ*_PE_ translating, via the Heisenberg uncertainty principle, to a sharper definition of *K*_*z*_^17,18^. As we will see below, in this case the ARPES signal provides the Fourier composition of the 2DEG wavefunctions. SX-ARPES on buried systems, challenged by photoelectron attenuation in the overlayers as well as a progressive reduction of photoexcitation cross-section of the valence band (VB) states with *hv*^19^, requires advanced synchrotron radiation sources delivering high photon flux (see Methods).

On the sample fabrication side, the 2DEG in GaN-HEMT heterostructures has until recently remained inaccessible to SX-ARPES because of the prohibitively large—of the order of 20–30 nm—depth of the AlGaN barrier layers. However, recent progress in molecular beam epitaxy (MBE) technology, in pursuit of yet higher operation frequencies of these devices, has allowed fabrication of heterostructures with ultrathin barrier layers of 3–4 nm^20–22^, which make them ideally suited to SX-ARPES. This has allowed direct **k**-space imaging of the fundamental electronic structure characteristics—the FS, electron dispersions, and the Fourier composition of wavefunctions—of the interfacial 2DEG in such heterostructures.

## Results

### Fabrication and basic electronic properties of GaN-HEMT heterostructures

**Fig. 1:**
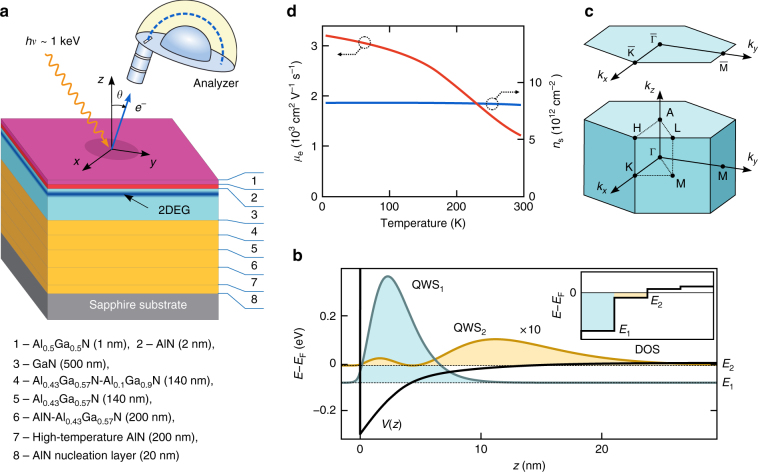
SX-ARPES experiment on the GaN-HEMT heterostructure. **a** Scheme of the epitaxial GaN-HEMT samples investigated by SX-ARPES. The photoelectron analyser detects the distribution $I_{\mathrm {PE}}(E_{\mathrm {k}},\vartheta )$ of the photoelectron kinetic energy *E*_k_ and emission angle $\vartheta$, which yield the binding energy *E*_B_ and momentum **k** back in the sample (corrected for the photon momentum *p* **=** *hv*/*c*) to produce the sought-for electron dispersions *E*(**k**). **b** *T*-dependence of *n*_s_ and $\mu_{e}$ obtained from Hall measurements. **c** Sketch of the electronic structure based on self-consistent solution of the 1D Poisson-Schrodinger equation. The quasi-triangular 1D potential *V*(*z*) confines two QWSs having different spatial localization of their electron density *n*^i^(*z*) (exaggerated by × 10 for the QWS_2_) centered at ~ 3 and ~ 12 nm for QWS_1_ and QWS_2_, respectively. The total three-dimensional DOS (insert) show steps characteristic of the 2D states. **d** Bulk BZ of GaN and 2D one of the GaN/AlGaN heterostructure

Our samples were grown on *c*-oriented sapphire substrate (see Methods). The 500 nm-thick Ga-polar GaN layer was grown on top of an AlGaN buffer layer required to suppress the crystal defects and promote growth of a smooth uniform film^23^. The GaN layer was overgrown by a barrier layer consisting of 2 nm of AlN and 1 nm of Al_0.5_Ga_0.5_N, see Fig. 1a. Hall-effect measurements on our samples, 1b, have found *n*_s_ ~ 8.2 × 10^12^ cm^−2^ almost constant through the temperature range *T* = 5-300 K that confirms the high quality of the fabricated structures.

A simplified electronic structure model of our GaN-HEMTs is shown in Fig. 1b. It was evaluated within the conventional envelope function approach (neglecting atomic corrugation) by self-consistent solutions of the one-dimensional (1D) Poisson–Schrödinger equations with the Dirichlet boundary conditions adjusted to reproduce the experimental *n*_s_ (for details see Supplementary Note 1). The effective 1D interfacial potential *V*(*z*) as a function of out-of-plane coordinate *z* confines two QWSs with different spatial localization. The QWS_1_ embeds larger partial *n*_s_ and is localized closer to the interface compared to the QWS_2_, which is shifted into the *V*(*z*) saturation region.

### FS: anisotropy and carrier concentration

A scheme of our SX-ARPES experiment is presented in Fig. 1a. The data were acquired in the *hv* region between 800 and 1300 eV where the interplay of photoelectron transmission through the barrier overlayer increasing with *hv* and photoexcitation cross-section decreasing with *hv* maximizes the QWS signal. In view of the even QWS wavefunction symmetry, we used *p*-polarization of the incident X-rays, which minimizes the geometry- and polarization-related matrix element effects, which distort the direct relation between ARPES intensities and Fourier composition of the QWS wavefunctions (see below).

**Fig. 2:**
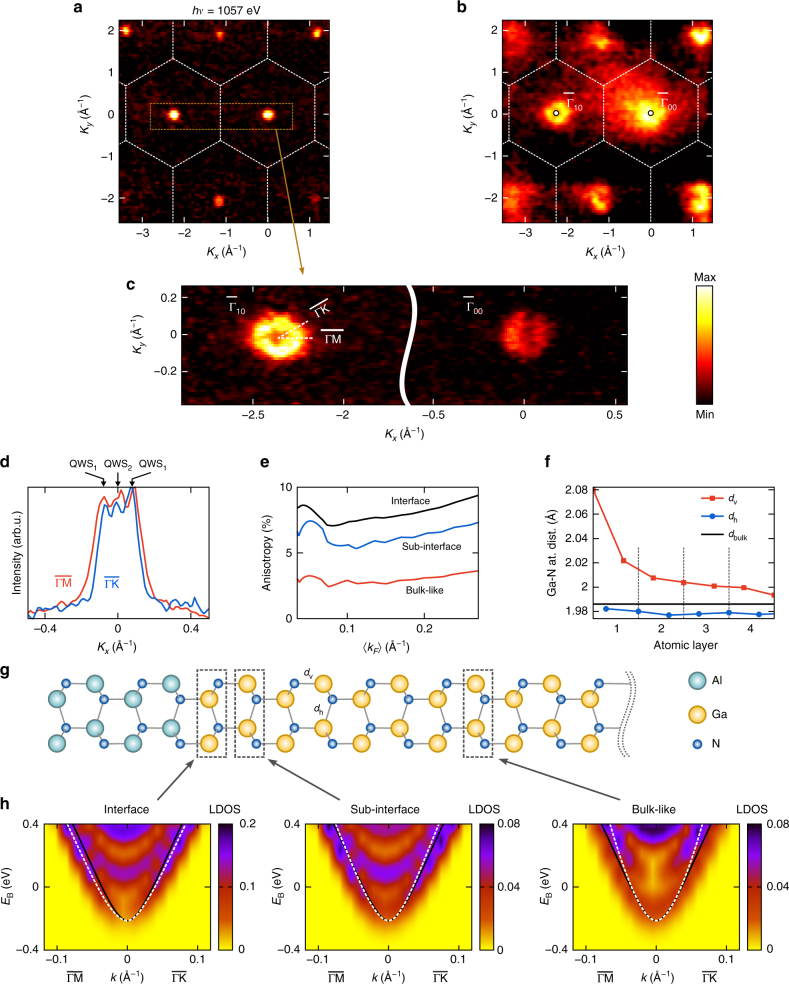
FS formed by the buried 2DEG. **a** Experimental FS formed by the 2DEG in comparison with **b** iso-*E*_B_ surface of the VB near the VBM. The FS appears as narrow electron pockets centered at the around the ${\bar{\mathrm \Gamma }}$-points, consistent with the CBM-derived character of the 2DEG. Both VB and FS maps reflect the C_6v_ symmetry of the GaN crystal lattice. **c** FS along the ${\bar{\mathrm \Gamma }}_{00} - {\bar{\mathrm \Gamma }}_{10}$ line acquired with high energy and angle resolution. **d** MDCs of the Fermi intensity around the ${\bar{\mathrm \Gamma }}_{10}$-point (derived from the high-statistics data in Fig. 4) identifying the tiny QWS_2_ and anisotropy of the QWS_1_ between the $\overline {{\mathrm{\Gamma M}}}$ and $\overline {{\mathrm{\Gamma K}}}$ azimuths with *A*_F_ ~ 12%. **e** Calculated *A*_F_ of QWS_1_ between $\overline {{\mathrm{\Gamma M}}}$ and $\overline {{\mathrm{\Gamma K}}}$ as a function of band filling characterized by < *k*_F_ > , for bulk GaN and for various heterostructure layers. **f** Relaxation of the Ga-N bond length as a function of depth and **g** u.c. used in the slab calculations. **h** **k**_//_-resolved LDOS for various heterostructure layers near the CBM with *E*_F_ adjusted to the experimental < *k*_F_ > and superimposed with the corresponding bulk *E*(**k**) along ГM and ГK (black lines). The QWS_1_ dispersion (white dashed in the bottom of the LDOS continuum) in the top GaN layers shows an asymmetry related to the interfacial atomic relaxation

The experimental FS map measured as a function of in-plane momentum **K**_*xy*_ = (*K*_*x*_,*K*_*y*_) at *hv* = 1057 eV (maximizing the QWS signal, see below) is presented in Fig. 2a. The FS formed by the QWSs in the HEMT channel appears as tiny circles whose **k**_//_ are located around the ${\bar{\mathrm \Gamma }}$-points of the heterostructure’s 2D Brillouin zone (BZ) shown in Fig. 1c. The location of the FS pockets coincides with the VB maxima (VBM), as seen in an iso-*E*_B_ map of the VB shown in Fig. 2b. In the direct band gap GaN, this location in **k**_//_ is consistent with the conduction band minimum (CBM)-derived character of the QWSs.

A high-resolution cut of the FS in Fig. 2c displays an external contour with a diameter of ~ 0.2 Å^−1^ manifesting the QWS_1_ and finite spectral weight in the middle, suggesting the presence of QWS_2_. The latter is confirmed by the momentum-distribution curves (MDCs) of the Fermi intensity around the ${\bar{\mathrm \Gamma }}_{10}$-points (Fig. 2d) where a weak structure in the middle signals the QWS_2_. Our observation of QWS_2_ is consistent with its recent detection via magnetotransport spectroscopy^24^. The presence of this state centered further away from the interface compared with the QWS_1_ indicates that *V*(*z*) in the GaN-HEMTs has a long-range saturated shape (see the model in Fig. 1b). The external Fermi intensity peaks in the *E*_F_-MDCs directly identify the Fermi momenta *k*_F_ of the QWS_1_.

Such a comprehensive **k**-space view of the buried QWSs, achieved with SX-ARPES, delivers two important observations. First, the experimental *E*_F_-MDCs reveal significant anisotropy of the FS characterized by a difference of the *k*_F_ values of 0.095 ± 0.006 Å^−1^ along the $\overline {{\mathrm{\Gamma M}}}$ azimuth and 0.085 ± 0.004 Å^−1^ along $\overline {{\mathrm{\Gamma K}}}$.These values were determined from the maximal gradient of the Fermi intensity^25^, which delivers accurate *k*_F_-values even when the bandwidth is comparable with the experimental resolution. The indicated statistical errors are the standard deviations of the *k*_F_ values over the measurement series and scaled with the Student’s *t*-distribution coefficients for a confidence interval of 76%. The corresponding *k*_F_ anisotropy factor $A_{\mathrm{F}} = \frac{{k_{\mathrm{F}}^{\overline {{\mathrm{\Gamma K}}} } - k_{\mathrm{F}}^{\overline {{\mathrm{\Gamma M}}} }}}{\langle{k_{\mathrm{F}}}\rangle}$, where $\langle k_{\mathrm{F}}\rangle = \left( {k_{\mathrm{F}}^{\overline {{\mathrm{\Gamma K}}} } + k_{\mathrm{F}}^{\overline {{\mathrm{\Gamma M}}} }} \right){\mathrm{/}}2$, measures in our case ~ 11%. Such a considerable FS anisotropy has been completely overlooked in previous macroscopic experimental studies without **k**-resolution.

Why does the 2DEG show such a pronounced planar anisotropy? Our simulations of electronic structure of the GaN-HEMTs (see Methods) used the standard density-functional theory (DFT), which is known to well describe excitation energies in GaN apart from the electron exchange-correlation discontinuity across the band gap approximated by the so-called scissors operator^26^. As the QWSs inherit their wavefunctions from the CBM of the bulk GaN (see below), we have started our analysis from band structure. The corresponding *A*_F_ in Fig. 2e is however negligible through the whole range of < *k*_F_ > defining the band filling. Next, we approached the electronic structure of our heterostructure system using GaN/AlN slab calculations with the unit cell (u.c.) shown in Fig. 2g. The atomic positions were relaxed under the constraint of the bulk GaN lateral lattice constants and symmetry. The resulting *c*-oriented Ga-N bond length *d*_v_ in Fig. 2f shows a significant increase toward the interface relative to the bulk value. The corresponding atomic displacement contributes to the piezoelectric polarization at the GaN/AlN interface. The layer-resolved electronic structure of this system was characterized by the **k**_//_-resolved layer density of states (LDOS) defined as, $\rho _z({\bf{k}}_{//},E) = \mathop {\int}\limits_\Omega {\mathrm{d}}x{\mathrm{d}}y{\mathop {\sum}\limits_n {\left| {\psi _n({\mathbf{r}},{\bf{k}}_{//},E)} \right|^2} }$, where **r** = (*x*,*y*,*z*), the summation includes all *n*-th electron states with wavefunctions $\psi _n$ available for given **k**_//_ and *E*, and the integration runs over the lateral unit cell Ω. Figure 2h shows the LDOS calculated near the CBM (the VB results are given in Supplementary Note 2) for the interface, sub-interface, and deep bulk-like GaN layers, where the bottom of the LDOS continuum corresponds to the QWS_1_. The corresponding *A*_F_ plots in 2e now show significant anisotropy, increasing toward the interface. At the experimental < *k*_F_ > the interface layer *A*_F_ is ~ 7%, which falls almost within the error bars of the experimental value. Finally, we performed the same LDOS calculations with the atomic coordinates in the slab fixed at the bulk values (without relaxation). *A*_F_ immediately returned to the negligible bulk values. This analysis suggests thus that the discovered 2DEG anisotropy in GaN-HEMTs is a purely interface effect caused by relaxation of atomic position near the GaN/AlN interface.

We note however that the predicted atomic relaxation is restricted to a few atomic layers next to the interface, and it is not clear why it should significantly affect the 2DEG, whose maximal density is located ~ 3 nm away from this region (Fig. 1b). In fact, even the state-of-art growth methods leave significant intermixing of Ga and Al atoms at the GaN/AlN interface^21^, resulting in a gradual variation of the lattice parameters over 1–3 nm from the interface. In the spirit of the entanglement between atomic relaxation and LDOS anisotropy, revealed by our computational analysis, this lattice distortion may cause significant electronic structure anisotropy extending into the 2DEG localization region.

Another important characteristic of the buried 2DEG is the experimental FS area which, by the Luttinger theorem^27^, is directly related to the *n*_s_ sheet carrier concentration. In our case, the area of the external QWS_1_ contour translates into partial $n_{\mathrm {s}}^{(1)}$ = (12.8 ± 1.4) × 10^12^ cm^−2^, and that of the internal QWS_2_ contour into $n_{\mathrm {s}}^{(2)}$ = (0.5 ± 0.4) × 10^12^ cm^−2^. We note that the emergence of two QWSs goes together with large *n*_s_ formed in the anomalously deep QW of the GaN-HEMTs. In our case, the QWS_2_ contributes only ~ 4% of the total *n*_s_ dominated by the QWS_1_. A significant difference of this ratio to that of 17% found in a cyclotron resonance study^28^ is explained by extremely high sensitivity of the QWS_2_ population to the interfacial QW depth in different samples. The total *n*_s_ in our case amounts to (13.3 ± 1.8) × 10^12^ cm^−2^, which is in fair agreement with *n*_s_ = 8.2 × 10^12^ cm^−2^ obtained by our Hall characterization, see Fig. 1d, in particular taking into account a small systematic overestimate of *k*_F_ introduced by the gradient method^25^.

### Momentum dependence of ARPES intensity: wavefunction character

Within the one-step theory of photoemission—see, e.g., ref. 29—the ARPES intensity is found as $I_{{\mathrm{PE}}} \propto \left| {\left\langle {f{\mathrm{|}}{\mathbf{A}} \cdot {\mathbf{p}}{\mathrm{|}}i} \right\rangle } \right|^2$, where $\left\langle {f|} \right.$ is the final and $\left. {|i} \right\rangle$ the initial states coupled through the vector potential **A** of the incident electromagnetic field and momentum operator **p**. Neglecting the experimental geometry and polarization effects, this expression simplifies to the scalar product $I_{{\mathrm{PE}}} \propto \left| {\left\langle {f{\mathrm{|}}i} \right\rangle } \right|^2$. For sufficiently high photon energies, $\left\langle {f|} \right.$ approximates a plane wave $e^{i{\mathbf{Kr}}}$ periodic in the in-plane *xy* direction and damped in the out-of-plane direction *z*, and the ARPES intensity appears^30,31^ as $I_{{\mathrm{PE}}} \propto \left| {\left\langle {e^{i{\mathbf{Kr}}}{\mathrm{|}}i} \right\rangle } \right|^2$. We will now apply this formalism to the QWS wavefunctions $\psi _{{\mathrm{QWS}}}$.

We will first analyze *I*_PE_ as a function of photoelectron **K**_*xy*_ in-plane momentum. If we represent $\psi _{{\mathrm{QWS}}}$ by Fourier expansion over 2D reciprocal vectors **g** as $\psi _{{\mathrm{QWS}}}\left( {\mathbf{r}} \right) = \mathop {\sum}\limits_{\mathbf{g}} {A_{{\mathbf{k}}_{xy} + {\mathbf{g}}}e^{i\left( {{\mathbf{k}}_{xy} + {\mathbf{g}}} \right){\mathbf{r}}_{xy}}}$, the plane-wave orthogonality will select from the sum only the component whose in-plane momentum **k**_*xy*_+**g** matches **K**_*xy*_ (corrected for the photon momentum *p* = *hv*/c), i.e., $I_{{\mathrm{PE}}}\left( {{\mathbf{K}}_{xy}} \right) \propto \left| {A_{{\mathbf{K}}_{{\boldsymbol{xy}}}}} \right|^2$. Therefore, the (*K*_*x*_,*K*_*y*_)-dependent ARPES maps in Fig. 2a–c visualize essentially the 2D Fourier expansion of $\psi _{{\mathrm{QWS}}}$^30,31^_._

**Fig. 3:**
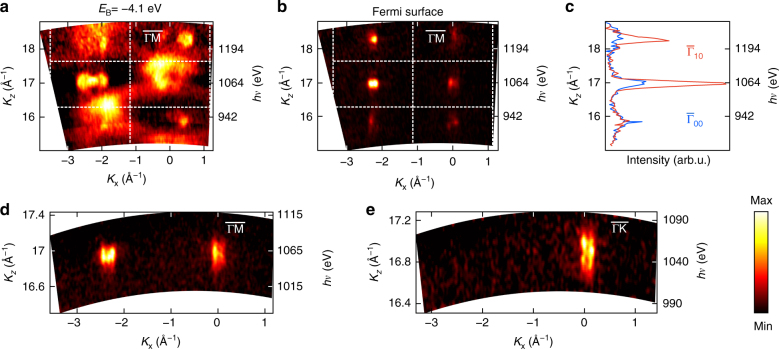
ARPES response of the 2DEG as a function of *K*_*z*_ momentum. ARPES intensity along the indicated azimuths is plotted as a function of *K*_*z*_ rendered from *hv* (the indicated *hv* values correspond to the ${\bar{\mathrm \Gamma }}$-point) **a** Iso-*E*_B_ map near the VBM, showing 3D contours of the VB states. **b** FS formed by the QWSs. **c** *E*_F_-MDCs at the ${\bar{\mathrm \Gamma }}_{00}$- and ${\bar{\mathrm \Gamma }}_{10}$-points, showing periodically oscillating response of the QWSs. Its peaks located in the Γ-points evidence that the QWSs inherit their wavefunction from the CBM of parent bulk GaN. Zoom-in of the FS at the ${\bar{\mathrm \Gamma }}_{00}$- and ${\bar{\mathrm \Gamma }}_{10}$-points along the $\overline {{\mathrm{\Gamma M}}}$ **d** and $\overline {{\mathrm{\Gamma K}}}$ **e** measured at around 1070 eV. The absence of its *K*_z_ dispersion confirms the 2D character of the QWSs

We will now analyze *I*_PE_ as a function of photoelectron *K*_*z*_ out-of-plane momentum varied in the experiment through *hv*. The corresponding iso-*E*_B_ map in (*K*_*x*_,*K*_*z*_) coordinates near the VBM is displayed in Fig. 3a. Its *K*_*z*_-dispersive contours demonstrate the three-dimensional (3D) character of the VB states inherited from bulk GaN. The FS in Fig. 3b formed by the QWSs demonstrates a different behavior however: the ARPES signal sharply increases whenever *K*_*z*_ approaches values of integer *G*_*z*_—corresponding to the Γ-points of the bulk BZ, but without any sign of *K*_*z*_ dispersion. The latter is emphasized by the zooms in Fig. 3d,e, where the QWSs form segments straight in the *K*_*z*_ direction. This pattern is characteristic of the 2D nature of the QWSs. The corresponding Fermi intensity is represented by *E*_F_-MDCs in Fig. 3c that show periodic oscillations peaked where *K*_*z*_ matches the Γ-points.

Why do the QWSs display such an ARPES response, periodically oscillating as a function of *hv*? The dependence of $\psi _{{\mathrm{QWS}}}$ on *z* for given **K**_*xy*_ is represented as $\psi _{{\mathrm{QWS}}}\left( z \right) = E\left( z \right) \cdot B_{k_z}\left( z \right)$, where the envelope function *E*(*z*) confines the oscillating Bloch wave $B_{k_z}\left( z \right)$ whose *k*_*z*_ momentum is adapted to the 3D crystal potential^32^. In our case, the first term is an Airy-like function embedded in the approximately triangular *V*(*z*) and the second one derives from bulk states of the GaN host. Inheriting ideas of early ARPES studies on surface states^32,33^, it can be shown^34^ that if we expand $B_{k_z}\left( z \right)$ over out-of-plane reciprocal vectors *G*_*z*_ of the 3D host lattice as $B_{{{k}}_z}(z) = \mathop {\sum}\limits_{G_z} {C_{G_z}e^{i\left( {k_z + G_z} \right)z}}$, then the *K*_*z*_ dependence of ARPES intensity for given **K**_*xy*_ appears as a sequence $I_{{\mathrm{PE}}}\left( {K_z} \right) \propto \mathop {\sum}\limits_{G_z} {\left| {C_{G_z}} \right|^2P\left( {K_z - (k_z + G_z)} \right)}$ of peaks $P\left( {K_z - (k_z + G_z)} \right)$ centered at *K*_z_ = *k*_*z*_ + *G*_*z*_. Physically, the ARPES intensity blows up whenever the photoelectron *K*_*z*_ hits a *k*_*z*_ + *G*_*z*_ harmonic of $\psi _{{\mathrm{QWS}}}$ to maximize the $\left\langle {f|} \right.$ and $\left| i \right\rangle$ scalar product. Amplitudes of the $I_{{\mathrm{PE}}}\left( {K_z} \right)$ peaks $\propto \left| {C_{G_z}} \right|^2$ image Fourier composition of the oscillating $B_{k_z}\left( z \right)$ term of $\psi _{{\mathrm{QWS}}}$ (modulated by *hv*-dependent photoelectron transmission through the AlGaN/AlN layer) and the peak shapes are related to the Fourier transform of the *E*(*z*) term weighted by $e^{ - \lambda _{{\mathrm{PE}}}z}$ ^34^.

Importantly, the experimental *K*_*z*_ dependence of the QWS signal in Fig. 3b exhibits peaks exactly at *k*_*z*_ + *G*_*z*_, corresponding to the Γ-point of bulk GaN. In combination with the (*K*_*x*_,*K*_*y*_) image in Fig. 2, where the QWS signal corresponds to the same Γ-point, this fact confirms that the $\psi _{{\mathrm{QWS}}}$’s are derived from the CBM states of bulk GaN. In a methodological perspective, such identification of the $\psi _{{\mathrm{QWS}}}$ character can be essential, e.g., for heterostructures of layered transition metal dichalcogenides, where the CB can include two or more valleys almost degenerate in energy but separated in **k**. The knowledge of the QWS character will then allow the predictive manipulation of the valley degree of freedom for potential valleytronics devices^35^.

We note that the common models of QWSs based on the 1D potential *V*(*z*), such as that in Fig. 1b, imply that their in-plane behavior $\psi _{{\mathrm{QWS}}}({\mathbf{r}}_{xy})$ is described by one single plane wave $e^{i{\mathbf{K}}_{xy}{\boldsymbol{r}}_{xy}}$ (i.e., one non-zero $A_{{\mathbf{K}}_{xy}}$ component) and out-of-plane behavior $\psi _{{\mathrm{QWS}}}(z)$ is identical to the smooth *E*(*z*) function. However, the experimental FS maps in Fig. 2a,c reveal numerous non-zero $A_{{\mathbf{K}}_{{\mathrm{xy}}}}$ spread through **k**-space and the $I_{PE}\left( {K_z} \right)$ oscillations numerous *k*_*z*_ + *G*_*z*_ harmonics. Accurate QWS models should therefore incorporate atomic corrugation of the interfacial potential in the in-plane and out-of-plane directions to form $\psi _{{\mathrm{QWS}}}$ as a confined Bloch wave.

The experimental distribution of high-energy ARPES intensity over sufficiently large volume of the (*K*_*x*_,*K*_*y*_,*K*_*z*_) space will in principle allow, notwithstanding the phase problem, a full reconstruction of $\Phi _{{\mathrm{QWS}}}(x,y,z)$ in all three spatial coordinates, similar to the reconstruction of molecular orbitals (see refs. 30,31, and references therein). This reconstruction will naturally incorporate full $\psi _{{\mathrm{QWS}}}$ including the envelope and Bloch-wave terms that goes beyond the common 1D models such as in Fig. 1b, describing the QWSs as free 2D electrons with empirical m* confined in the *z* direction. More accurate models of the GaN-HEMTs should replace free electrons by Bloch ones, naturally incorporating atomic corrugation.

### Band dispersions: effective mass

**Fig. 4:**
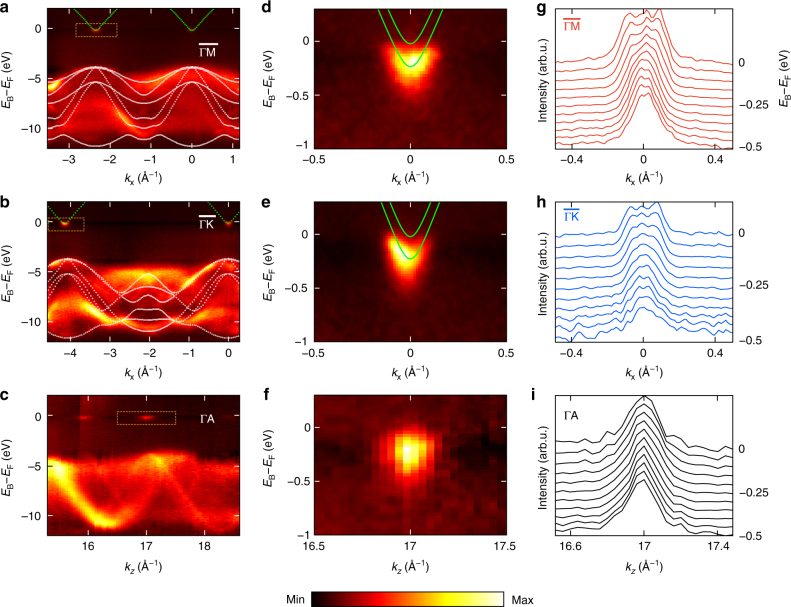
Band dispersions of the buried 2DEG. **a**–**c** Experimental band structure measured at *hv* = 1066 eV for the **a** ΓK and **b** ΓM directions of the bulk BZ (superimposed with calculated *E*(**k**) of bulk GaN, white dashed lines) and under variation of *hv* for **c** ΓA, cf. Fig. 3. The CBM-derived QWSs appear above the VB continuum. **d**–**f** Zoom-in image of the QWSs around the ${\bar{\mathrm \Gamma }}_{10}$-point (green lines schematize their dispersions fitting the experimental *k*_F_). **g**–**i** (Normalized) MDCs around the ${\bar{\mathrm \Gamma }}_{10}$-point for a series of *E*_B_ through the QWS bandwidth. The difference between the $\overline {{\mathrm{\Gamma M}}}$ and $\overline {{\mathrm{\Gamma K}}}$ dispersions manifests planar anisotropy of the 2DEG, and the absence of *k*_*z*_ dispersion confirms its 2D character

Experimental band dispersions in GaN-HEMTs shown in Fig. 4 were measured along $\overline {{\mathrm{\Gamma}}{\mathrm{M}}}$ (a) and $\overline {{\mathrm{\Gamma}}{\mathrm{K}}}$ (b) at *hv* = 1066 eV bringing *K*_*z*_ to the Γ-point of the bulk BZ. Non-dispersive ARPES intensity coming from the AlN and AlGaN layers is suppressed in these plots by subtracting the angle-integrated spectral component. The CBM-derived QWSs appear as tiny electron pockets above the VB dispersions of GaN. Their energy separation from the VBM is consistent with the GaN fundamental band gap of ~ 3.3 eV. Whereas the VB dispersions are broadened in *E*_B_ primarily because of band bending in the QW region, the QWS dispersions stay sharp. This confirms their 2D nature insensitive to band bending as well as their localization in the deep defect-free region in GaN, spatially separated from the defect-rich GaN/AlN interface region, the fundamental operational principle of the HEMTs delivering high $\mu _{\mathrm {e}}$. Fig. 4c,f,i show the experimental dispersions as a function of *k*_z_. Whereas clear dispersion of the VB states manifests their 3D character, the QWS are flat in *K*_*z*_.

A zoom-in of the QWS dispersions along the $\overline {{\mathrm{\Gamma M}}}$ and $\overline {{\mathrm{\Gamma K}}}$ azimuths is shown in Fig. 4d,e with the corresponding MDC in Fig. 4g,h. Whereas the outer contour of these dispersions corresponds to the QWS_1_, the significant spectral weight in the middle is due to the QWS_2_. Following the *k*_F_ anisotropy discussed above, a parabolic fit of the QWS_1_ dispersions yields m* values of (0.16 ± 0.03)*m*_0_ along the $\overline {{\mathrm{\Gamma M}}}$ azimuths and (0.13 ± 0.02)*m*_0_ along $\overline {{\mathrm{\Gamma K}}}$ (*m*_0_ is the free-electron mass), which thus differ from each other by ~ 22%.

Our SX-ARPES experiment presents thus a direct evidence of the planar FS and m* anisotropy in GaN-HEMTs. This effect was overlooked in previous studies, because the optics methods are **k**-integrating and quantum oscillation techniques lose their **k**-resolution in the 2D case. Magnetotransport experiments give only an indirect information on the 2DEG’s m*^10–12^, which is conventionally^36,37^ assumed to be isotropic. We conjecture that further progress of SX-ARPES on energy resolution will push data analysis from merely band structure to one-electron spectral function *A*(*ω*,**k**), which will inform, e.g., the interaction of electrons with other quasiparticles such as the phonon-plasmon-coupled modes.^38^

### Implications for the transport properties

How will the observed lateral anisotropy of the 2DEG electronic structure affect the transport properties? Naively, one might think that it would directly translate into an anisotropy of the electrical conductivity. However, fundamental linear response considerations attest that any physical properties such as conductivity described by a second-order tensor with C_6v_ symmetry must be scalar, i.e., in the linear (low-field) regime, conductivity in the hexagonal lattice of GaN must be isotropic (Supplementary Note 3). On the other hand, this restriction is lifted for the nonlinear (high-field) regime where conductivity can become anisotropic. A canonical example of such a crossover is *n*-doped Ge.^39,40^ Although its FS is anisotropic, cubic symmetry of the Ge lattice results in isotropic low-field conductivity. However, with an increase of the electric field, conductivity along the $\left\langle {001} \right\rangle$, $\left\langle {011} \right\rangle$, and $\left\langle {111} \right\rangle$ crystallographic directions develops differently. The GaN-HEMTs can be easily driven into the nonlinear regime where electronic current saturates due to electron scattering on longitudinal optical (LO) phonons^41,42^. To reach the LO phonon energy, lighter electrons should gain a larger drift velocity *V*_sat_. Therefore, larger *V*_sat_ and thus saturation current *I*_sat_ should be expected in the directions of smaller m*.

**Fig. 5:**
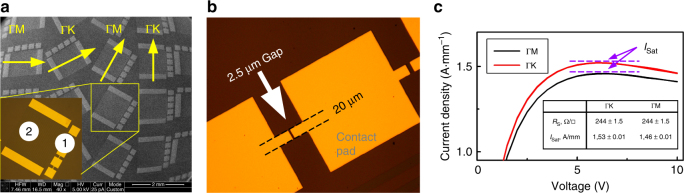
Electron transport measurements. **a** Test modules, oriented in four directions (scanning electron microscope images, slightly distorted due to large view area): TLM modules for the contact resistance measurements (marked 1 in the inset) and ‘resistor’ modules for *R*_s_ determination (marked 2). The yellow arrows indicate the TLM azimuthal orientation. **b** Region of the TLM modules with the channel length 2.5 μm used for the *I*–*V* measurements (optical microscope image). **c** *I*–*V* characteristics for the $\overline {{\mathrm{\Gamma M}}}$ and $\overline {{\mathrm{\Gamma K}}}$ azimuths. The inset table summarizes the mean *R*_s_ and *I*_sat_ values for different azimuths. Although *R*_s_ is essentially isotropic, higher *I*_sat_ for the $\overline {{\mathrm{\Gamma K}}}$ azimuth in comparison with $\overline {{\mathrm{\Gamma M}}}$ indicates smaller m* of electrons moving along $\overline {{\mathrm{\Gamma K}}}$

We have examined low- and high-field conductivity in our GaN-HEMT heterostructures using samples essentially identical to the ARPES ones, but with the Al_0.45_Ga_0.55_N layer thickness increased to 15 nm, to prevent a 2DEG degradation during longer sample handling in air. Hall measurements showed *n*_s_ = 2 × 10^13^ cm^−2^, *μ*_e_ = 1150 cm^2^ V^−1^ s, and sheet resistance *R*_s_ = 240 Ω sq^−1^ for these samples. The fabricated test modules were oriented at four different angles (0°, 30°, 60°, 90°) with respect to the substrate to promote current flow along the $\overline {{\mathrm{\Gamma M}}}$ and $\overline {{\mathrm{\Gamma K}}}$ azimuths (Fig. 5a,b). Results of the transport measurements presented in Fig. 5c show, as expected, isotropic low-field *R*_s_. However, the *I*_sat_ characteristic of the high-field regime is clearly anisotropic: 1.53 ± 0.01 A ×  mm^−1^ along $\overline {{\mathrm{\Gamma K}}}$ and 1.46 ± 0.01 A × mm^-1^ along $\overline {{\mathrm{\Gamma M}}}$ (Fig. 5c). As a consistency check, the modules rotated by 60° with respect to each other showed the same *I*_sat_ values, in accordance with hexagonal symmetry of the GaN electronic structure. These results on the previously overlooked *I*_sat_ anisotropy demonstrate that m* along $\overline {{\mathrm{\Gamma K}}}$ is smaller compared to $\overline {{\mathrm{\Gamma M}}}$, as predicted by our ARPES results.

## Discussion

Our direct **k**-space imaging of the fundamental electronic structure characteristics—FS, band dispersions and occupancy, and Fourier composition of wavefunctions—of the 2DEG formed in high-frequency GaN-HEMTs with ultrathin barrier layer makes a quantitative step compared to conventional optics and magnetotransport experiments. We discover, in particular, significant planar anisotropy of the 2DEG band dispersions caused by piezoelectrically active relaxation of atomic position near the GaN/AlN interface. This effect is found to manifest itself in nonlinear electron transport properties as anisotropy of the saturation drift velocity and current. Our findings suggest a positive use of the crystallographic orientation to improve these high-power characteristics of GaN-HEMTs. Furthermore, our **k**-space image of the Fourier composition of the 2DEG wavefunctions calls for extension of the conventional 1D models of semiconductor interfaces to 3D ones based on the Bloch-wave description naturally incorporating atomic corrugation. The fundamental knowledge about GaN-HEMTs achieved in our work as well as new device simulation methods can clarify the physical limits of these heterostructures, and finally push their reliable operation into the near-THz range. Methodologically, we have demonstrated the power of the synchrotron radiation based technique of SX-ARPES with its enhanced probing depth and sharp definition of the full 3D **k** for the discovery of previously obscured properties of semiconductor heterostructures. Our results complement previous applications of SX-ARPES to buried oxide interfaces^7^ and magnetic impurities in semiconductors^43^ and topological insulators^44^, which used elemental and chemical-state specificity of this technique achieved with resonant photoemission. In a broader perspective, our methodology arms the heterostructure growth technology with means to directly control the fundamental **k**-space parameters of the electronic structure, thereby delivering optimal transport and optical properties of the fabricated devices. Complementary to imaging of non-equilibrium electron motion in spatial coordinates,^45^ we can envisage an extension of our experimental methodology to pump–probe experiments using X-ray free-electron laser sources, which will image the electron system evolution in **k**-space during transient processes in electronic devices.

## Methods

### Sample fabrication

The GaN-HEMT heterostructures embedding a 2DEG were grown on *c*-oriented sapphire substrate in a SemiTeq STE3N MBE-system equipped with an ammonia (NH_3_) nitrogen source. The buffer layer growth adopted the procedure described in ref. 23. Before deposition, the substrate was annealed during 1 h and then nitrided for 40 min with 30 sccm NH_3_ at 850 °C. The following growth was carried out with 200 sccm NH_3_. Deposition started with 20 nm AlN layer, grown at 1050 °C. The following 200 nm AlN layer was grown at 1120 °C with Ga used as a surfactant. Then a gradient junction to Al_0.43_Ga_0.57_N with a thickness of ~ 250 nm was achieved by a gradual decrease of the substrate temperature down to *T* *=* 830 °C, followed by 140 nm of growth at constant *T*. Next, a second gradient junction to Al_0.1_Ga_0.9_N with a thickness of ~ 140 nm was formed by reducing *T* of the Al effusion cell. Then a 500 nm GaN layer was grown. The growth was finished by deposition of a barrier layer consisting of 2 nm AlN and 1 nm Al_0.5_Ga_0.5_N for the ARPES experiments, and 1 nm AlN and 15 nm Al_0.45_Ga_0.55_N for measurements of transport properties. Hall effect characterization was carried out in magnetic fields up to 40 kG. The magnetic-field dependences were measured in both the standard Hall and van der Pauw geometries. All  transport measurements were carried out at the Resource Center of Electrophysical Methods (Complex of NBICS-Technologies of Kurchatov Institute).

### SX-ARPES experiments

Raw SX-ARPES data were generated at the Swiss Light Source synchrotron radiation facility (Paul Scherrer Institute, Switzerland). The experiments have been carried out at the SX-ARPES endstation^46^ of the ADRESS beamline^47^, delivering high photon fluxes up to 10^13^ photons × s^−1^ × (0.01% BW)^−1^. With the actual experimental geometry, *p*-polarized incident X-rays selected electron states symmetric relative to the $\overline {{\mathrm{\Gamma M}}}$ and $\overline {{\mathrm{\Gamma K}}}$ azimuths. The projection *K*_*x*_ of the photoelectron momentum was directly measured through the emission angle along the analyser slit, *K*_*y*_ is varied through tilt rotation of the sample, and *k*_*z*_ through *hv*. The experiments were carried out at 12 K to quench the thermal effects reducing the coherent **k**-resolved spectral component at high photoelectron energies^48^. The combined (beamline and analyzer) energy resolution was ~ 150 meV and the angular resolution of the PHOIBOS-150 analyzer was 0.07°. The X-ray spot size in projection on the sample was 30 × 75 μm^2^, which allowed us to control spatial homogeneity of our samples. Charging effects were not detected due to the small thickness of the AlGaN barrier layer.

### Electronic structure calculations

First-principles calculations for bulk GaN have been carried out in the DFT framework as implemented in the pseudopotential Quantum Espresso code^49^ using ultrasoft pseudopotentials. The electron exchange-correlation term was treated within the Generalized Gradient Approximation using the Perdew–Burke–Ernzerhof functional. Self-consistent calculations for bulk GaN were performed with the plane-wave cutoff energy 60 Ry and **k**-space sampling over a grid of 10 × 10 × 5 points in the BZ and corrected with the scissors operator to reproduce the experimental band gap. Calculations for the GaN-HEMT heterostructure used a 1 × 1 slab geometry with the supercell including 18 u.c. of GaN in the middle between 3 u.c of AlN at each end, Fig. 2g. Atomic coordinates in the supercell were relaxed, but imposing the lateral u.c. of bulk GaN until the Hellmann–Feynman forces on each atom were < 30 meV Å^−1^. The plane-wave cutoff energy was 25 Ry with a **k**-grid of 10 × 10 × 1 points. The Gaussian window for LDOS calculations was set to 0.05 eV.

### Transport measurements

Raw transport data were generated at the Kurchatov Institute. To measure linear and nonlinear transport properties of the GaN-HEMT heterostructures, two types of test modules with low-resistance regrown ohmic contacts^50,51^ were formed. Details on processing can be found in ref. 51. The first type modules were conventional transmission line measurement (TLM) modules with a channel width of 20 μm and channel lengths of 2.5, 10, 20, and 40 μm (marked 1 in Fig. 5a). These modules were used to determine contact resistance, which was found to be 0.15 Ω mm. Also *I*–*V* curves were measured at the smallest gaps (2.5 μm length channels, see Fig. 5b) of 24 such TLM modules (6 modules per each of four directions) in DC mode. The voltage sweep time (1 ms per point with a voltage step of 0.5 V) was chosen to be small enough to suppress sample heating effects as judged by the absence of hysteresis in the forward and backward voltage scans as well as repeatability of the *I*–*V* curves with a sweep time reduction. The second type “resistor” modules (marked 2 in Fig. 5a) were arrays of 1 mm long and 20 μm wide stripes (25 stripes per module separated by 20 μm mesa isolation) with contact pads on each side. These modules had negligible contact resistance and were used for precise measurement of the 2DEG low-field conductivity in different directions.

### Data availability

Derived data supporting the findings of this study are available from the corresponding author on request. The SX-ARPES data were processed using the custom package MATools available at https://www.psi.ch/sls/adress/manuals.

## Electronic supplementary material

Supplementary Information

Peer Review Report
