# Supplementary material for: k-space imaging of anisotropic 2D electron gas in GaN/GaAlN high-electron-mobility transistor heterostructures
Source: Nat Commun. 2018 Jul 11;9:2653. doi: 10.1038/s41467-018-04354-x (PMC6041315; doi:10.1038/s41467-018-04354-x)
Supplement: Supplementary file 1 — Supplementary Information [file 41467_2018_4354_MOESM1_ESM.pdf]

Supplementary Information

k-space imaging of anisotropic 2D electron gas in GaN/GaAlN high-electron-mobility transistor heterostructures

by Lev et al.

Supplementary Note 1
1D model of the GaN-HEMT heterostructure

To describe 2DEG localized at the GaN/AlGaN interface, we employ a simple unipolar Poisson-Schrodinger self-consistent model. The equilibrium electron density in 2DEG is given by

$$n(\mathbf{r}) = \int \mathrm{d}E\; f(E)\rho(E,\mathbf{r})\,, \tag{1}$$

where $f(E)$ is the Fermi-Dirac distribution and $\rho(E,\mathbf{r})$ is the local density of states in 2DEG that is determined by solving the Schrodinger equation.

The ideal system is translation invariant in the 2DEG plane ($\mathbf{r}_{||}$), and the wavefunctions can be written in the factorized form

$$\Psi_{n,\mathbf{k}_{||}}(\mathbf{r}) = e^{\mathrm{i}\mathbf{k}_{||}\mathbf{r}_{||}}\psi_n(z), \quad \mathbf{r} \equiv (\mathbf{r}_{||}, z), \;\; \mathbf{k}_{||} \equiv (k_x, k_y)\,, \tag{2}$$

where $\mathbf{k}_{||}$ is the in-plane electron momentum, and *n* is the quantum number that characterizes discrete quantum levels in the quantum well which is formed in the perpendicular direction (*z*). The wavefunction $\psi_n(z)$ satisfies the following Schrodinger equation:

$$\left[\frac{\hbar^2}{2}\left(\frac{k_x^2}{m_x(z)} + \frac{k_y^2}{m_y(z)} - \frac{\mathrm{d}}{\mathrm{dz}}\frac{1}{m_z(z)}\frac{\mathrm{d}}{\mathrm{dz}}\right) + V_{\mathrm{CBM}}(z) + V(z)\right]\psi_n(z) = E_n(\mathbf{k}_{||})\psi_n(z)\,, \tag{3}$$

where $V_{\mathrm{CBM}}(z)$ is the potential profile of the conduction band minimum, and $V(z)$ is the Hartree electrostatic potential energy due to other electrons that is to be determined self-consistently.

The problem can be greatly simplified if electron motion in the 2DEG plane can be decoupled from the out-of-plane. To this aim, we assume [1] that the in-plane effective masses $m_x(z)$ and $m_y(z)$ are independent of *z*. This approximation is justified due to the very small penetration of $\psi_n(z)$ into the AlGaN barrier layer. Then (3) reduces to

$$\left[-\frac{\hbar^2}{2}\left(\frac{\mathrm{d}}{\mathrm{dz}}\frac{1}{m_z(z)}\frac{\mathrm{d}}{\mathrm{dz}}\right) + V_{\mathrm{CBM}}(z) + V(z)\right]\psi_n(z) = E_n\psi_n(z)\,, \tag{4}$$

with the energy $E_n(\mathbf{k}_{||})$ given by

$$E_n(\mathbf{k}_{||}) = E_n + \frac{\hbar^2}{2m_x}k_x^2 + \frac{\hbar^2}{2m_y}k_y^2\,. \tag{5}$$

After the Schrodinger equation (4) is solved, the density of states in (1) can be computed:

$$\rho(E,\mathbf{r})=\rho(E,z)=2\sum_n|\psi_n(z)|^2\int\frac{\mathrm{d}^2\mathbf{k}_{\parallel}}{(2\pi)^2}\delta\left(E-E_n(\mathbf{k}_{\parallel})\right)=\frac{\sqrt{m_x m_y}}{\pi\hbar^2}\sum_n|\psi_n(z)|^2\,\theta\left(E-E_n\right), \tag{6}$$

where the factor 2 stands for the double spin degeneracy, $\theta(x)$ is the Heaviside step function, and the $\psi_n(z)$ are assumed to be normalized. Then the electron density is

$$n(\mathbf{r})=n(z)=k_{\mathrm{B}}T\frac{\sqrt{m_x m_y}}{\pi\hbar^2}\sum_n|\psi_n(z)|^2\ln\left[1+\exp\left(\frac{E_{\mathrm{F}}-E_n}{k_{\mathrm{B}}T}\right)\right], \tag{7}$$

where *T* is the 2DEG temperature, and E_{F} is the Fermi level.

The electrostatic potential $V(z)=-e\phi(z)$ is computed from the Poisson equation:

$$-\frac{\mathrm{d}}{\mathrm{dz}}\left(\varepsilon(z)\frac{\mathrm{d}\phi(z)}{\mathrm{dz}}\right)=4\pi\rho(z) \tag{8}$$

with the full charge density $\rho(z)$ given by

$$\rho(z)=e\left[N_d^+(z)-n(z)+\sum_k\sigma_k\delta(z-z_k)\right]. \tag{9}$$

Here $N_d^+(z)$ is the concentration of ionized residual donors in GaN, and the last term describes the contribution of spontaneous and piezoelectric polarization charges with the surface density σ_k localized at the heterostructure interface at positions z_k.

For the Poisson equation we assume the Dirichlet boundary condition at the outer AlN surface and the zero Neumann boundary condition deep into the GaN buffer layer:

$$\phi(0)=\phi_{\mathrm{s}},\ \phi'(L)=E_z(L)=0\,. \tag{10}$$

The unknown surface potential ϕ_{s} is adjusted so that the value of the total electron density in the quantum well

$$n_{\mathrm{s}}=\int\mathrm{dz}\;n(z) \tag{11}$$

matches the ARPES and Hall measurements results.

The Gummel-type self-consistent iterations between eqs. (4) and (8) have poor convergence. It can be significantly improved if (8) is turned into a non-linear Poisson equation with a potential-dependent full charge density functional $\rho[\phi(z)]$ that is obtained using the first-order perturbation theory for (4). Details of this procedure can be found in [2].

The exchange-correlation potential $V_{\mathrm{xc}}(z)$ in the local density approximation can also be included in (4), however, it leads to just a few meV correction of the quantum levels [3] and is ignored here.

Supplementary Note 2
Slab calculations for the GaN/AlN interface

Atomic relaxation and electronic structure of the GaN/AlN interface was simulated with slab calculations (for details see Methods of the main text). The supercell used in these calculations, Fig. 1 (*top*), used a geometry of 1x1 u.c. of bulk GaN in the lateral direction and 18 u.c. in the perpendicular direction which were confined by 3 u.c of AlN on each side. The symmetric supercell geometry and thus potential distribution was essential to suppress spurious electron states. The atomic positions were relaxed under constraint of the lateral u.c. symmetry and basis parameters. They are characterized by significant variations of the *c*-oriented Ga-N bond length in the perpendicular direction (see Fig. 2 in the main text).

k-resolved layer density of states (LDOS) [4,5] calculated through the valence and conduction bands for different atomic planes in the GaN/AlN supercell is shown in Fig. 1 (*bottom*). It is compared with the corresponding bulk GaN band dispersions calculated for k_z = 0 and adjusted in energy to match the LDOS peaks at the VBM and CBM. In fact, the bulk is represented by already the 8th atomic layer in the middle of the supercell, which recaptures the bulk bond lengths and band structure of the bulk.

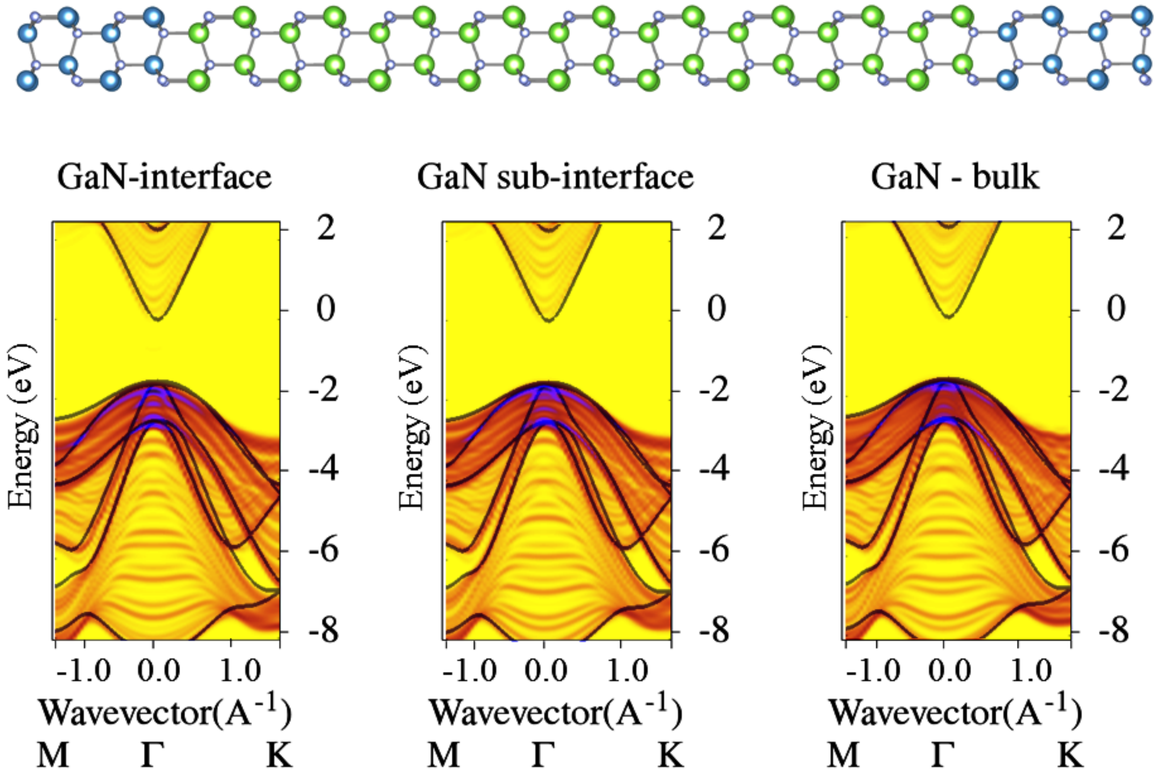

Supplementary Figure 1. (*top*) The supercell used in the calculations; (*bottom*) **k**-resolved LDOS for the GaN layer at the interface with AlN, for the 3-rd layer, and for the 8-th layer representing the GaN bulk. The superimposed solid curves are the bulk GaN bandstructure.

A zoom-in of the **k**-resolved LDOS from Fig. 1 in the CBM region is given in Fig. 2 of the main text. The LDOS outer contour corresponds to the QWS_1. Importantly, the QWS_1 dispersion in the interface layer demonstrates significant ΓM/ΓK asymmetry that progressively reduces towards the bulk. k_F values for the A_F factor quantifying the asymmetry (see the main text) were determined from parabolic fit of the QWS_1 dispersion in LDOS along ΓM and ΓK within ± 0.3 eV around the Fermi level. The evaluated A_F as a function of band filling is presented in Fig. 2 of the main text.

Finally, we have performed the same self-consistent electronic structure calculations where the atomic coordinates were set to the bulk GaN lattice parameters without relaxation. The calculated LDOS in Fig. 2 demonstrates that in this case the interfacial ΓM/ΓK asymmetry reduces to its insignificant bulk values. This simulation supports our interpretation of the 2DEG anisotropy as resulting from relaxation of the interfacial atoms.

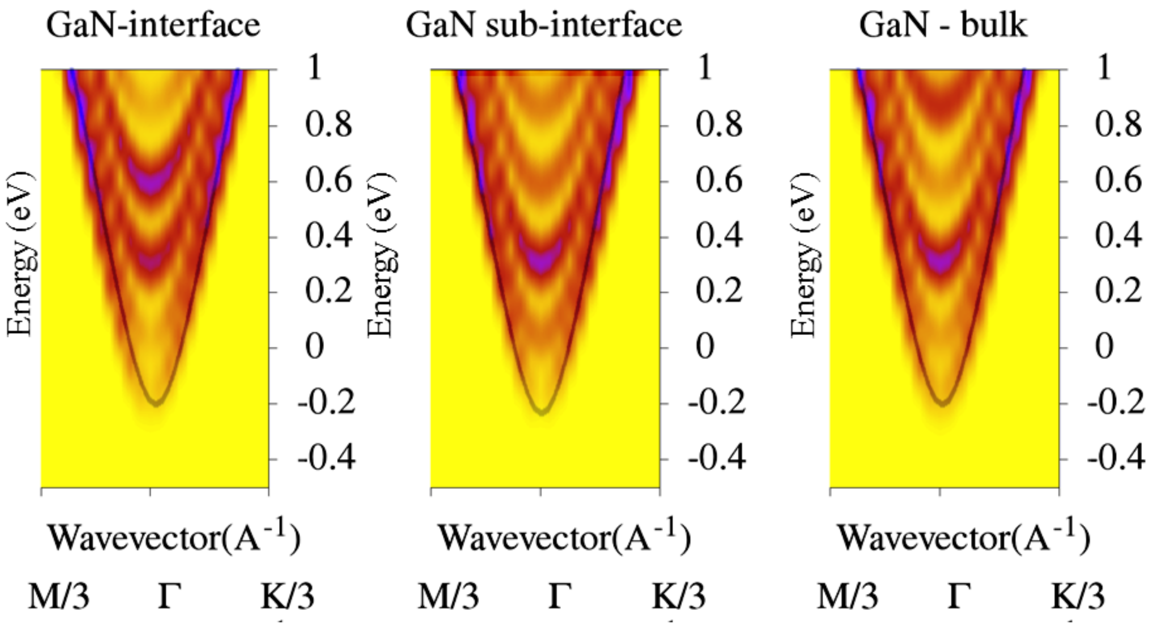

Supplementary Figure 2. The same LDOS in the CBM region as in Fig. 1 but calculated without relaxation of the interface atoms. The ΓM/ΓK asymmetry essentially disappears.

Supplementary Note 3
Crystal symmetry and electron conductivity

In this section we consider the crystal symmetry effect on linear (low-field) conductivity of crystals with anisotropic electronic structure.

The mathematics and discussions below can be in general form found in several textbooks (see, for example, [6]). However, implications of these principles to transport properties may be not obvious for non-specialists in transport properties of solid state systems. We have therefore compiled here these ideas in a focused form in order to save non-specialist readers from extensive literature search and calculations.

Applying external electric field $\vec{E}$ results in appearance of current with density $\vec{j} = \vec{j}(\vec{E})$. At low electric fields their relation is linear, and as such it has the following properties:

$$\vec{j}(\alpha\vec{E}) = \alpha\vec{j}(\vec{E}), \quad \text{(homogeneity)} \tag{1}$$

$$\vec{j}(\vec{E}_1 + \vec{E}_2) = \vec{j}(\vec{E}_1) + \vec{j}(\vec{E}_2), \quad \text{(additivity)}. \tag{2}$$

For any in-plane unit basis $(\vec{e}_x, \vec{e}_y)$ (we are interested in the fields and currents in the 2DEG plane) we have $\vec{E} = E_x\vec{e}_x + E_y\vec{e}_y$; using the properties (1), (2), we obtain:

$$\vec{j}(\vec{E}) = E_x\vec{j}(\vec{e}_x) + E_y\vec{j}(\vec{e}_y), \tag{3}$$

or, in the matrix form:

$$\begin{pmatrix} j_x \\ j_y \end{pmatrix} = \begin{pmatrix} \sigma_{xx} & \sigma_{xy} \\ \sigma_{yx} & \sigma_{yy} \end{pmatrix} \begin{pmatrix} E_x \\ E_y \end{pmatrix} = \hat{\sigma} \begin{pmatrix} E_x \\ E_y \end{pmatrix}, \text{ or } j_\alpha = \sigma_{\alpha\beta}E_\beta, \tag{4}$$

where $\hat{\sigma}$ is the conductivity tensor, α and β run through the x and y coordinates, and the $\vec{j}(\vec{e}_x)$ and $\vec{j}(\vec{e}_y)$ vectors is decomposed in our 2D basis as $\vec{j}(\vec{e}_x) = \sigma_{xx}\vec{e}_x + \sigma_{xy}\vec{e}_y$, $\vec{j}(\vec{e}_y) = \sigma_{yx}\vec{e}_x + \sigma_{yy}\vec{e}_y$. Now we will consider how the symmetry of a crystal restricts the form of tensors representing its physical properties. According to the Neumann's symmetry principle, if a crystal is invariant with respect to certain symmetry operations, all its physical properties must also be invariant with respect to this symmetry operations. In other words, the symmetry group of any physical property of a crystal must include the whole point symmetry group of the crystal. In particular, the latter means that if $\hat{A}$ is a symmetry transformation operator, then in the linear case $\hat{A}(\vec{j}(\vec{E})) = \vec{j}(\hat{A}(\vec{E}))$.

Let us consider two types of in-plane symmetry operations in Cartesian coordinate system: rotation and reflection. The matrix of rotation around the origin by angle θ is

$$\hat{R} = \begin{pmatrix} \cos\theta & \sin\theta \\ -\sin\theta & \cos\theta \end{pmatrix} \tag{5}$$

If system is symmetric with respect to rotation by angle θ, the two following matrix products must be equal:

$$\hat{R}\hat{\sigma}=\begin{pmatrix}\sigma_{xx}\cos\theta+\sigma_{xy}\sin\theta & \sigma_{yx}\cos\theta+\sigma_{yy}\sin\theta\\ -\sigma_{xx}\sin\theta+\sigma_{xy}\cos\theta & -\sigma_{yx}\sin\theta+\sigma_{yy}\cos\theta\end{pmatrix} \quad \textbf{(6)}$$

$$\hat{\sigma}\hat{R}=\begin{pmatrix}\sigma_{xx}\cos\theta-\sigma_{xy}\sin\theta & \sigma_{xx}\sin\theta+\sigma_{xy}\cos\theta\\ \sigma_{yx}\cos\theta-\sigma_{yy}\sin\theta & \sigma_{yx}\sin\theta+\sigma_{yy}\cos\theta\end{pmatrix} \quad \textbf{(7)}$$

Equating the corresponding matrix components, we obtain

$$\begin{cases}\sigma_{xx}\sin\theta=\sigma_{yy}\sin\theta,\\ \sigma_{xy}\sin\theta=-\sigma_{yx}\sin\theta.\end{cases} \quad \textbf{(8)}$$

In particular, from (8) it follows that any $\theta:\sin\theta\neq 0$ immediately yields $\sigma_{xx}=\sigma_{yy}$.
Now consider a reflection, for simplicity, with respect to the abscissa axis. The corresponding transformation matrix

$$\hat{T}=\begin{pmatrix}1 & 0\\ 0 & -1\end{pmatrix}, \quad \textbf{(9)}$$

renders the $(0,E_y)$ vector to $(0,-E_y)$ one, leaves the $(E_x,0)$ vector unchanged. Equating the $\hat{\sigma}\hat{T}$ and $\hat{T}\hat{\sigma}$ products, one finds that $\sigma_{xy}=\sigma_{yx}=0$, and therefore

$$\hat{\sigma}=\begin{pmatrix}\sigma_{xx} & 0\\ 0 & \sigma_{yy}\end{pmatrix}. \quad \textbf{(10)}$$

As we see, symmetry with respect to rotation by an angle $\theta:\sin\theta\neq 0$, combined with the in-plane axial reflection symmetry, results in a diagonal conductivity tensor where the diagonal components are equal. I.e., the linear conductivity tensor is a scalar in this case. We should mention that the same considerations are valid for the case of (0001) plane of C_6 (hexagonal) point group crystals, (0001) plane of C_{3v} point group crystals, (001) plane of cubic crystals, and other symmetrical systems fulfilling the above requirements.

Supplementary References

1. Bastard, G. *Wave mechanics applied to semiconductor heterostructures.* EDP Sciences, 1992.

2. Trellakis, A., Galick, A. T., Pacelli, A., Ravaioli, U. Iteration scheme for the solution of the two-dimensional Schrodinger-Poisson equations in quantum structures. *J. Appl. Phys.* **81**, 7880-7884 (1997).

3. Jogai, B. Free electron distribution in AlGaN-GaN heterojunction field-effect transistors. *J. Appl. Phys.* **91**, 3721-3729 (2002).

4. Inglesfield, J. E. Surface electronic structure. *Rep. Prog. Phys.* **45**, 223-284 (1982).

5. Shikin, A. M. *et al*. Effect of spin–orbit coupling on atomic-like and delocalized quantum well states in Au overlayers on W(110) and Mo(110). *New J. Physics* **15**, 125014 (2013).

6. Nye, J.F. *Physical Properties of Crystals: Their Representation by Tensors and Matrices.* Clarendon Press, 1985.
