# Supplementary material for: k-space imaging of anisotropic 2D electron gas in GaN/GaAlN high-electron-mobility transistor heterostructures
Source: Nat Commun. 2018 Jul 11;9:2653. doi: 10.1038/s41467-018-04354-x (PMC6041315; doi:10.1038/s41467-018-04354-x)
Supplement: Supplementary file 2 — Peer Review Report [file 41467_2018_4354_MOESM2_ESM.pdf]

Reviewers' comments:

Reviewer #1 (Remarks to the Author):

This work reports measurements of the band structure of the two-dimensional electron gas (2DEG) at the GaN/AlGaN heterostructure, widely used today as a basic component of high electron mobility transistors (HEMTs). The authors use heterostructures with ultrathin AlGaN barrier layers of 3-4 nm, and angle-resolved photoemission spectroscopy in the soft-X-ray regime (SX-ARPES), capable of directly probing the photoelectrons emitted from the buried ultrathin interface. The measurements thus provide the momentum-resolved Fermi surface and bands of the interfacial 2DEG –and also of the valence band of the underlying bulk GaN. The main new finding of the work, compared to more typical and momentum-insensitive optical and magneto-transport studies, and to standard theoretical approaches to the physics of the 2DEG in GaN-HEMTs, is a planar anisotropy of the Fermi surface (of the order of 12% between the Fermi momenta along Gamma-K and Gamma-M) and, concomitantly, of the bands and effective mass of the 2DEG in the GaN/AlGaN interface. To understand this, the authors perform density-functional theory (DFT) simulations of the electronic structure of the GaN-HEMTs, and find that the observed anisotropy is purely an interface effect caused by the relaxation of the atomic positions near the GaN/AlN interface. This finding should help improving theories and engineering of HEMTs based on such heterostructures.

I find that the data in this paper is technically sound, and together with the developed analysis, provides convincing evidence for most of its conclusions, excepting the determination of the 2DEG's wavefunction (see below). These results should be important to specialists in high-mobility semiconductor heterostructures.

However, there is at least one important point that must be addressed before reaching a conclusion regarding publication in Nature Communications: The paper claims (both at the introduction and outlook) to have determined, through the measured SX-ARPES data, the momentum-dependent wavefunctions of the quantum-well states in the GaN/AlGaN interface. However, such wavefunctions are not determined at all. What the authors actually infer from the SX-ARPES data are two rather straightforward facts:

1) The measured (kx, ky)-dependent ARPES intensity (in-plane Fermi surface of the 2DEG, Fig. 2a) shows the periodicity of the GaN lattice. This can be essentially understood in terms of Bloch theorem: the 2DEG's electronic structure (hence its spectral function, measured by ARPES) has the periodicity of the underlying lattice. I don't see the need to exaggerate this through an elaborate explanation based on a 2D Fourier decomposition of the 2DEG wavefunctions, as currently presented in the paper (lines 151-162 and Supplementary Material). Such discussion, in its present form, is in fact very confusing. Maybe what the authors actually mean by an "analysis of the in-plane ARPES intensity" is the description of the less trivial modulations in intensity superimposed to the periodicity required by Bloch theorem? Such modulations come from: (i) effects of the experimental geometry, which the authors actually neglect without justification (Supplementary Material), but should nevertheless discuss: matrix elements due to light polarization and relative orientation between the sample and the analyzer slits, and how all these change when the sample is rotated to obtain a 2D Fermi-surface map; and (ii) final-state effects, as a cut at fixed photon energy corresponds to a spherical cap in momentum space –hence, not all the associated surface-projected Gamma points will match with bulk Gamma points, where final states for photon absorption are available.

2) The Fermi surface of the 2DEG along k_z, measured by varying the photon energy, is non dispersive, as expected for a quasi-2D system. However, the ARPES intensity around EF shows a strong kz-dependence, periodically peaking at k_z values corresponding to the Gamma points of bulk GaN. Such modulation in intensity, observed and discussed in many previous ARPES works on 2D electron systems, is again basically due to the availability of empty final bulk states for the

photon absorption process, as these states have the periodicity of the bulk lattice along k_z. This is the essence of the discussion presented in the paper (lines 173-188). But here again, I find such discussion rather technical and confusing for the non-specialist. The paper would gain in pedagogy by simplifying it. Moreover, at the end of the discussion, neither the wavefunctions of the electron quantum well states, nor the envelope function of the confining potential, are really determined – contrary to what the introduction and outlook sections of the paper lead to believe. Such misleading claims should therefore be revised or eliminated.

Other minor issues that need to be corrected are the correspondence between the labels of Fig. 2 and the description of the data in the text. For instance:

* Line 121: “The corresponding A_f in Fig. 2f”… refers probably to Fig. 2e?
* Line 124: “The unit cell shown in Fig. 2h”… should rather be Fig. 2g?
* Line 125: “The resulting c-oriented Ga-N bond length dv in Fig. 2g”… or rather Fig. 2f?
* Line 131: where is Fig. 2i?
* Line 133: “The corresponding A_f plots in 2f”… should rather be 2e?

Reviewer #2 (Remarks to the Author):

In addition to this, I would support publication in Nature Communications provided the authors address the following concerns and comments:
1. For Fig.1A, in principle, the APRES measurement only detects the AlGaN/AlN/GaN interface of the first three layers. It remains unclear why there is a need to grow the complex 5 layers before the growth of 500 nm GaN;
2. For Fig. 1C, Is there any experimental evidence (such as from SdH oscillation of the authors own sample) to support on the model that there are two quantum well states confined in the well?
3. Across the manuscript, there are scattered comparison of the GaN-2DEG with those of oxide 2DEGs, such as resonant photoemission and the electronic phase separation, I think it is very important and relevant, in the outlook part, to highlight the universal difference between the GaN-2DEG with the ZnMgO/ZnO 2DEG (A. Tsukazaki et al. Science 315, 1388, 2007) and the 3d oxide 2DEG in CaZrO3/SrTiO3 (Chen et al. Nano Lett. 15, 1894, 2015), three of the typical 2DEGs formed by strain-induced polarization.

4. In the first paragraph of the introduction, the fundamental operational principle of the HEMTs is called “modulation doping”. Is this also applied in the top GaN interface detected by the ARPES?

Reviewer #3 (Remarks to the Author):

This paper reports the momentum-resolved electronic structure of the 2DEG at the buried interface AlGaN/GaN, whose high carrier mobility makes it an indispensable material in high-frequency applications like microwave communications. The authors reveal the anisotropy of the effective mass of the 2DEG and the physical origin of the anisotropy, which may be helpful for further increasing the carrier mobility, and further enhancing the performance of 2DEGs based devices in practical applications, even to THz-range. These experimental findings are enabled by recent advances in experimental technologies: (1) the soft X-ray ARPES using high resolution, high brightness beam lines, and (2) MBE growth technique that reduces the capping layer to a few nano-meters. This manuscript is well written, and provides new insights on the wave function of 2DEG at the AlGaN/GaN, which could be critical to advances in 2DEG related technologies. I would recommend this paper to be published on Nature Communications, if the authors could address the following issues:
(1) The FWHM of photoemission along Γ-A is around 0.2Angstrom^-1 (the bottom panel of Fig. 4c), which corresponds to an envelope function E(z) with FWHM around 3nm. Since this FWHM of

3nm is much smaller than the extension of quantum well states in Fig. 1c, the width of E(z) is mainly determined by the photoelectron escape depth λ, and thus the photoemission signal is contributed by part of the 2DEG located in about 3nm of thickness near the interface at the GaN side. This thickness range contains more atomic layers than those illustrated in Fig.2g. However, according to the authors' calculation, the most prominent anisotropy is at the interface layer, but still weaker than the experimental value. Moreover, the functioning part of the 2DEG that has high mobility should be away from the interfacial layer that may give more scattering. Can the model of relaxation of interfacial atomic position still explain the observed anisotropy from around 3nm of thickness? If the anisotropy comes from the atomic relaxation very near the interface, is this anisotropy still an important property of the 2DEG that stays a little bit away from the interface? And how much does such an anisotropy contribute to the transport properties?

(2) The anisotropy of Fermi momentum is observed at a rather small Fermi surface. The momentum separation of the measured Fermi momenta is extremely sensitive to the momentum location of the photoemission cut. For example, the cut across Gamma shows largest Fermi crossing, while the cut off Gamma would be smaller even for an isotropic Fermi momentum. It is important to prove that the momentum cuts Γ-M and Γ-K in Fig.2d are indeed through the Brillouin zone center before reaching the conclusion of anisotropic Fermi momentum. For example, the binding energies of band bottom along Gamma-M and Gamma-K should be identical and should be the minimum compared with cuts of slightly varied momenta. Do the two cuts measured meet the conditions? Is the observed anisotropy six-fold symmetric?

(3) Could the effective mass be estimated from the measured band dispersion if Fig.4b? Are the effective masses consistent with those of previous reports and that of CBM of GaN?

REPLIES TO THE REVIEWER REPORTS

Reviewer #1:

This work reports measurements of the band structure of the two-dimensional electron gas (2DEG) at the GaN/AlGaN heterostructure, widely used today as a basic component of high electron mobility transistors (HEMTs). The authors use heterostructures with ultrathin AlGaN barrier layers of 3-4 nm, and angle-resolved photoemission spectroscopy in the soft-X-ray regime (SX-ARPES), capable of directly probing the photoelectrons emitted from the buried ultrathin interface. The measurements thus provide the momentum-resolved Fermi surface and bands of the interfacial 2DEG –and also of the valence band of the underlying bulk GaN. The main new finding of the work, compared to more typical and momentum-insensitive optical and magneto-transport studies, and to standard theoretical approaches to the physics of the 2DEG in GaN-HEMTs, is a planar anisotropy of the Fermi surface (of the order of 12% between the Fermi momenta along Gamma-K and Gamma-M) and, concomitantly, of the bands and effective mass of the 2DEG in the GaN/AlGaN interface. To understand this, the authors perform density-functional theory (DFT) simulations of the electronic structure of the GaN-HEMTs, and find that the observed anisotropy is purely an interface effect caused by the relaxation of the atomic positions near the GaN/AlN interface. This finding should help improving theories and engineering of HEMTs based on such heterostructures.

I find that the data in this paper is technically sound, and together with the developed analysis, provides convincing evidence for most of its conclusions, excepting the determination of the 2DEG's wavefunction (see below). These results should be important to specialists in high-mobility semiconductor heterostructures.

We thank the reviewer for the appreciation of the scientific merits and importance of our work. We believe that the revised manuscript, now including explicit connection of the ARPES results with non-linear device performance of the GaN-HEMTs, stands even higher scientific standards.

However, there is at least one important point that must be addressed before reaching a conclusion regarding publication in Nature Communications: The paper claims (both at the introduction and outlook) to have determined, through the measured SX-ARPES data, the momentum-dependent wavefunctions of the quantum-well states in the GaN/AlGaN interface. However, such wavefunctions are not determined at all.

The reviewer is absolutely correct, and what we observe in the ARPES experiment is just moduli of Fourier components of the QW wavefunctions. Strictly speaking, exact recovery of the wavefunctions requires knowledge of (1) phases of the Fourier components not directly observed in the experiment, and (2) complete ARPES dataset covering all excitation energies and the whole ***k****-space. The wavefunction can in principle be recovered from our dataset using some iterative algorithms [see, for example, the cited Puschnig et al., Science 326, 702 (2009); P. Kliuiev et al., New J. Phys. 18, 093041 (2016)] but this is obviously beyond the scope of the present work. Following the reviewer's criticism on this point, through*

the whole paper we have replaced the claimed determination of wavefunctions by more exact determination of their Fourier composition.

What the authors actually infer from the SX-ARPES data are two rather straightforward facts:

1) The measured (kx, ky)-dependent ARPES intensity (in-plane Fermi surface of the 2DEG, Fig. 2a) shows the periodicity of the GaN lattice. This can be essentially understood in terms of Bloch theorem: the 2DEG's electronic structure (hence its spectral function, measured by ARPES) has the periodicity of the underlying lattice. I don't see the need to exaggerate this through an elaborate explanation based on a 2D Fourier decomposition of the 2DEG wavefunctions, as currently presented in the paper (lines 151-162 and Supplementary Material). Such discussion, in its present form, is in fact very confusing. Maybe what the authors actually mean by an "analysis of the in-plane ARPES intensity" is the description of the less trivial modulations in intensity superimposed to the periodicity required by Bloch theorem?

The ***k****-space periodicity of the ARPES signal is of course trivial, merely reflecting the 2D periodicity of the our heterostructure. We analyze however not periodicity but intensity modulations of the ARPES signal as a function of* $\mathbf{k}_{//}$ *and hv, reflecting Fourier composition of wavefunctions in the in-plane and out-of-plane directions. We have rephrased the text to make this point clearer. The ARPES intensity considerations are actually crucial to prove that the 2DEG wavefunctions strongly deviate from trivial confined plane waves, because otherwise the ARPES intensity would have shown only one single peak where final-state* ***K*** *had matched the single Fourier component of these waves.*

Such modulations come from: (i) effects of the experimental geometry, which the authors actually neglect without justification (Supplementary Material), but should nevertheless discuss: matrix elements due to light polarization and relative orientation between the sample and the analyzer slits, and how all these change when the sample is rotated to obtain a 2D Fermi-surface map;

Our ARPES experiment used p-polarization of incident X-rays which, with the 2DEG wavefunctions having even symmetry, introduces only minor matrix element dependence on the emission angle. We have specified this important point in the revised version.

and (ii) final-state effects, as a cut at fixed photon energy corresponds to a spherical cap in momentum space –hence, not all the associated surface-projected Gamma points will match with bulk Gamma points, where final states for photon absorption are available.

The reviewer is absolutely right that fixed photon energy corresponds to a sphere of out-of-plane momentum K_z *depending on in-plane one* $\mathbf{K}_{xy}$*. However, at high photon energy the radius of this sphere, roughly proportional to* $\sqrt{hv}$*, becomes very large, and* K_z *can be considered fairly constant across the presented Fermi surface maps as a function of* $\mathbf{K}_{xy}$*. Furthermore, the Fermi surface maps as a function of (*K_x*,*K_z*) are rendered from the emission angle and photon energy coordinates using the explicit kinematic formulas including photon momentum, as specified in the manuscript.*

2) The Fermi surface of the 2DEG along k_z, measured by varying the photon energy, is non dispersive, as expected for a quasi-2D system. However, the ARPES intensity around EF shows a strong kz-

dependence, periodically peaking at k_z values corresponding to the Gamma points of bulk GaN. Such modulation in intensity, observed and discussed in many previous ARPES works on 2D electron systems, is again basically due to the availability of empty final bulk states for the photon absorption process, as these states have the periodicity of the bulk lattice along k_z. This is the essence of the discussion presented in the paper (lines 173-188). But here again, I find such discussion rather technical and confusing for the non-specialist. The paper would gain in pedagogy by simplifying it.

The discussion regarding physics of photon energy dependence, periodically peaking at k_z harmonics of the confined Bloch wave, was indeed too technical for the non-specialist. In the revised version we have much simplified it. Interestingly enough, we have discussed this seemingly trivial physics with many ARPES specialists, and for many of them this was totally unknown. Therefore, we have removed from the manuscript the lengthy supplementary reviewing the corresponding Fourier transform formalism, and posted it at arXiv [http://arxiv.org/abs/1801.07505 (2018)]. We feel that this rearrangement helps the reader focus on the particular GaN-HEMT electronic structure.

Moreover, at the end of the discussion, neither the wavefunctions of the electron quantum well states, nor the envelope function of the confining potential, are really determined –contrary to what the introduction and outlook sections of the paper lead to believe. Such misleading claims should therefore be revised or eliminated.

We certainly agree on this point. The initial claims of the wavefunctions determination were actually made in the sense of determination of their Fourier composition, in order to simplify the story for the non-specialist. However, this phrasing can be quite an oversimplification. As we said before, in the revised version we have replaced it by more exact phrasing of the Fourier composition determination.

Other minor issues that need to be corrected are the correspondence between the labels of Fig. 2 and the description of the data in the text. For instance:

* Line 121: “The corresponding A_f in Fig. 2f”… refers probably to Fig. 2e?

* Line 124: “The unit cell shown in Fig. 2h”… should rather be Fig. 2g?

* Line 125: “The resulting c-oriented Ga-N bond length dv in Fig. 2g”… or rather Fig. 2f?

* Line 131: where is Fig. 2i?

* Line 133: “The corresponding A_f plots in 2f”… should rather be 2e?

We have fixed these typos in the revised manuscript. We sincerely thank the reviewer for the constructive critique and valuable suggestions that have helped us significantly improve the manuscript in its scientific content and readability. We believe that the revised manuscript, reinforced by new high-statistics ARPES datasets and results of non-linear transport measurements on the GaN-HEMT heterostructures confirming the ARPES predictions, stands the high scientific standards of Nature Communications.

Reviewer #2:

The manuscript of Lev et al. reports the synchrotron ARPES of the high mobility 2DEG in GaN-based transistor heterostructures, one of the typical high mobility 2DEGs which are formed by strain-induced polarization. They observed planar anisotropic electron Fermi surface and anisotropy in effective mass. The results are interesting. My main complaint is that whether there is detectable anisotropy in the transport properties of the grown structure?

We thank the reviewer for the appreciation of the scientific merits and importance of our work, and in particular for this point that has stimulated our in-depth investigations of transport anisotropy. A whole new section and another Supplementary on transport experiment and their analysis have been introduced into the revised manuscript. Here we want to emphasis some important points.

On the sample preparation side, in a short period of time since we received the reviewer reports we have designed and fabricated modules tailored to the transport anisotropy measurements with precisely controlled geometry and uniformity. We have used our proprietary technology to fabricate "regrown contacts" to the 2DEG, whereas the conventional "alloyed contacts" failed to produce reproducible results. The samples were grown with a thicker barrier layer compared to the ARPES ones to ensure uniformity and stability of the 2DEG during handling and transport measurements in atmospheric conditions.

Our transport experiments directly addressed the reviewer's question on whether the Fermi surface and effective mass anisotropy propagates to transport. Our answer is yes and no. For linear (low-field) transport our measurements have found isotropic conductivity. This seemingly surprising result stems from fundamental linear response principles applied to the hexagonal GaN lattice. As these considerations may be surprising for non-experts in physics of electronic transport, we have compiled them in another Supplementary chapter. For non-linear (high-field) transport these principles are lifted, however, and we have indeed detected significant anisotropy of saturation drift velocity, in agreement with the ARPES findings. This link between ARPES and electron transport is a major upgrade of our work, directly propagating to technology of GaN-based devices.

In addition to this, I would support publication in Nature Communications provided the authors address the following concerns and comments:

1. For Fig.1A, in principle, the APRES measurement only detects the AlGaN/AlN/GaN interface of the first three layers. It remains unclear why there is a need to grow the complex 5 layers before the growth of 500 nm GaN;

The need to grow complex underlayers prior to the GaN deposition comes from the common problem of nitride technology: due to high cost and low availability of single-crystal GaN and AlN substrates, nitride heterostructures are commonly grown on SiC, sapphire, and silicon substrates. Their extremely large lattice mismatch to GaN (about 13%) complicates direct epitaxy of GaN layers. The research team of I. Akasaki, awarded by the Nobel Prize in Physics in 2014, was the first to break through this difficulty by growing a buffer layer between GaN and sapphire substrate. Since then development of the buffer

growth techniques have been in the core of GaN technology. Our samples were grown with a proprietary buffer structure developed in NRC "Kurchatov institute". Its technical details and underlying physics are described in a recent paper in Journal of Applied Physics cited in the revised manuscript.

2. For Fig. 1C, Is there any experimental evidence (such as from SdH oscillation of the authors own sample) to support on the model that there are two quantum well states confined in the well?

Our ARPES data directly visualizes the two QW states as well-resolved peaks in the momentum distribution curves of Fermi intensity in Fig. 2d. In the context of our work, it has been most important to elucidate the ability of ARPES to detect such subtle electronic structure features. Studies of the two QW states with SdH oscillations in GaN/AlGaN heterostructures are actually standard experiments reported in numerous works, for example, in the cited paper by Mishra et al., and we have left them beyond the scope of the present work. However, we fully agree with the referee that the SdH oscillations can in future be a good supplementary method to compare with the ARPES results.

3. Across the manuscript, there are scattered comparison of the GaN-2DEG with those of oxide 2DEGs, such as resonant photoemission and the electronic phase separation, I think it is very important and relevant, in the outlook part, to highlight the universal difference between the GaN-2DEG with the ZnMgO/ZnO 2DEG (A. Tsukazaki et al. Science 315, 1388, 2007) and the 3d oxide 2DEG in CaZrO3/SrTiO3 (Chen et al. Nano Lett. 15, 1894, 2015), three of the typical 2DEGs formed by strain-induced polarization.

We thank the reviewer for mentioning these oxide systems that share with GaN/AlGaN the same ideas of creating 2DEGs through spontaneous and strain-induced polarization. We have included the corresponding references in the Introduction and highlighted the universal difference between the semiconductor and oxide systems in n_s *different by orders of magnitude.*

4. In the first paragraph of the introduction, the fundamental operational principle of the HEMTs is called “modulation doping”. Is this also applied in the top GaN interface detected by the ARPES?

In the introduction we mention that "A characteristic property of HEMTs based on wurtzite GaN/AlGaN heterostructures is the accumulation of large sheet carrier concentration $n_s \sim 10^{13}$ cm^{-2} *… without intentional doping of the barrier layer". This also applies to our samples. Actually, n-doping of the barrier layer was widely used at the beginning of GaN-HEMTs, but a series of works in late 90-s have established the dominant role of spontaneous and piezoelectric polarization in the 2DEG formation. This makes the barrier doping unnecessary.*

Reviewer #3:

This paper reports the momentum-resolved electronic structure of the 2DEG at the buried interface AlGaN/GaN, whose high carrier mobility makes it an indispensable material in high-frequency applications like microwave communications. The authors reveal the anisotropy of the effective mass of the 2DEG and the physical origin of the anisotropy, which may be helpful for further increasing the carrier mobility, and further enhancing the performance of 2DEGs based devices in practical applications, even to THz-range. These experimental findings are enabled by recent advances in experimental technologies: (1) the soft X-ray ARPES using high resolution, high brightness beam lines, and (2) MBE growth technique that reduces the capping layer to a few nano-meters. This manuscript is well written, and provides new insights on the wave function of 2DEG at the AlGaN/GaN, which could be critical to advances in 2DEG related technologies.

We thank the reviewer for the appreciation of the scientific merits and importance of our work. We believe that the revised manuscript, now including explicit connection of the ARPES results with non-linear device performance of the GaN-HEMTs, is even more important for the scientific community.

I would recommend this paper to be published on Nature Communications, if the authors could address the following issues:

(1) The FWHM of photoemission along Γ-A is around 0.2Angstrom^-1 (the bottom panel of Fig. 4c), which corresponds to an envelope function E(z) with FWHM around 3nm. Since this FWHM of 3nm is much smaller than the extension of quantum well states in Fig. 1c, the width of E(z) is mainly determined by the photoelectron escape depth λ, and thus the photoemission signal is contributed by part of the 2DEG located in about 3nm of thickness near the interface at the GaN side. This thickness range contains more atomic layers than those illustrated in Fig.2g. However, according to the authors' calculation, the most prominent anisotropy is at the interface layer, but still weaker than the experimental value. Moreover, the functioning part of the 2DEG that has high mobility should be away from the interfacial layer that may give more scattering. Can the model of relaxation of interfacial atomic position still explain the observed anisotropy from around 3nm of thickness? If the anisotropy comes from the atomic relaxation very near the interface, is this anisotropy still an important property of the 2DEG that stays a little bit away from the interface?

This is an excellent point. In the revised version we address it in a short paragraph explaining that even the state-of-art growth methods leave significant intermixing of Ga and Al atoms at the GaN/AlN interface resulting in a gradual variation of lattice parameters over 1-3 nm from the interface. In spirit of the entanglement between atomic relaxation and LDOS anisotropy revealed by our computational analysis, this lattice distortion may cause significant electronic structure anisotropy reaching out to the 2DEG localization region. After all, our ARPES observation of the 2DEG lateral anisotropy is fully confirmed by non-linear transport measurements (see below).

And how much does such an anisotropy contribute to the transport properties?

We particularly thank the reviewer for this point. It has motivated us to complement our ARPES results by extensive transport measurements (see Methods). Unexpectedly, they have revealed a beautiful picture of crossover between isotropic electron transport in the linear (low-field) regime and anisotropic in the non-linear (high-field) regime. In the revised manuscript, we have devoted to these results another section of the text and figure. Briefly, fundamental linear response considerations for the hexagonal lattice of GaN (described in detail in new Supplementary 3) predict that in the linear regime conductivity must be isotropic even despite the anisotropic Fermi surface and effective mass. This restriction is lifted for the non-linear regime where conductivity can become anisotropic. Indeed, we have experimentally verified that low-field conductivity of our GaN-HEMT heterostructures is isotropic, but the saturation current, characteristic of the high-field regime, shows clear anisotropy. Our finding of the lateral 2DEG anisotropy suggests thus improvement of high-power characteristics of GaN-HEMTs by proper crystallographic alignment of the HEMT channel.

(2) The anisotropy of Fermi momentum is observed at a rather small Fermi surface. The momentum separation of the measured Fermi momenta is extremely sensitive to the momentum location of the photoemission cut. For example, the cut across Gamma shows largest Fermi crossing, while the cut off Gamma would be smaller even for an isotropic Fermi momentum.

For 2D states – that do not disperse as a function of out-of-plane momentum K_z – the momentum separation in in-plane momentum K_{xy} should be identical, at least if not distorted by exotic matrix element effects. In any case, we measured the Fermi surface cuts in different Γ-points with different photon energies for in order to maintain the same K_z that delivers maximal ARPES intensity (see the periodic K_z-dependence of ARPES intensity in Fig. 3b,c).

It is important to prove that the momentum cuts Γ-M and Γ-K in Fig.2d are indeed through the Brillouin zone center before reaching the conclusion of anisotropic Fermi momentum. For example, the binding energies of band bottom along Gamma-M and Gamma-K should be identical and should be the minimum compared with cuts of slightly varied momenta. Do the two cuts measured meet the conditions? Is the observed anisotropy six-fold symmetric?

Due to the smallness of the observed Fermi surface anisotropy we have performed the experiment with extreme care. First, the center of the Fermi surface in the K_y direction was determined from ARPES intensity maps measured in steps as fine as 0.05^o in the corresponding sample angle setting, which is nearly twice smaller that the analyzer angular acceptance in this direction. Second, the cuts along ΓM and ΓK, measured at different azimuthal settings of the sample, were brought to the same position on the detector, in order to exclude any influence of potential imperfection of the analyzer angular scale. Third, the angular scale at this particular position for given retarding ratio and pass energy was verified by measurements of angle dispersion using a slit array with a separation of 1°. We have certainly verified that the band bottom energy along the ΓM and ΓK was the same, and at its minimum under variations of the K_y momentum. The measurements performed at three azimuthal settings for ΓM and three for ΓK showed k_F values identical within the experimental errors to confirm six-fold symmetry of these values. Finally, for one ΓM and one ΓK azimuthal setting the k_F measurements were performed in seven different points over the sample to exclude any sample inhomogeneity effects. Accuracy of the obtained k_F values

was estimated as their standard deviation through individual measurements multiplied by the Student's coefficient corresponding to the standard 2σ confidence interval of 76%. With such a careful data acquisition and data analysis procedures we are confident in reliability of our results.

(3) Could the effective mass be estimated from the measured band dispersion if Fig.4b? Are the effective masses consistent with those of previous reports and that of CBM of GaN?

We thank the reviewer for pointing out this significant caveat in our work. Based on the second-order fit of the experimental 2DEG dispersions, the effective mass determination is actually very sensitive to the experimental statistics. Additional series of high-statistics SX-ARPES data has been acquired and refined data processing algorithms used in order to evaluate the explicit effective mass values added into the revised version. We sincerely thank the reviewer for the constructive critique and valuable suggestions that have helped us significantly improve the manuscript in its scientific content and readability. We believe that the revised manuscript, reinforced by new high-statistics ARPES datasets and results of non-linear transport measurements on the GaN-HEMT heterostructures connected with the ARPES predictions, stands the high scientific standards of Nature Communications.

REVIEWERS' COMMENTS:

Reviewer #1 (Remarks to the Author):

The revised version of the manuscript and supplementary material fulfill the requests and concerns I formulated. The additional new data on the low- and high-field conductivity in the GaN-HEMT heterostructures, showing the onset of an anisotropic saturation current at high field, strengthens the conclusions drawn from the SX-ARPES measurements. I therefore recommend publication of this work in Nature Communications.

Reviewer #2 (Remarks to the Author):

Dear Editor,

The revised manuscript is much improved. The SX-APRES data are better explained and the new evidence in transport anisotropy of the saturation drift velocity and current further supports their interesting finding. I view this paper will not only be important for developing new strategies to improve the performance of GaN-HEMTs, but also contributes to understand interfacial 2DEGs of general interest. I recommend the publication of this manuscript for Nature communications.

Reviewer #3 (Remarks to the Author):

In responding to the reviewers' comments, the revised manuscript by Lev et al. has been substantially improved. Especially the new section on transport experiment provides further support to their conclusions. I recommend acceptance of the current manuscript for publication in Nature Communications after considering some minor suggestions.
Some parts of the main text are too detail for the readers to grab the main ideas smoothly. The authors use a large paragraph (lines 60~74) to explain the advantages of the SXARPES technique, including some aspects like chemical resolution is not relevant to this study. The authors may considering shorten this part, and move the details to the methods section.

Dear Dr. Thomas,

In the final review round, we have received the following responses:

Reviewer #1 (Remarks to the Author):

The revised version of the manuscript and supplementary material fulfill the requests and concerns I formulated. The additional new data on the low- and high-field conductivity in the GaN-HEMT heterostructures, showing the onset of an anisotropic saturation current at high field, strengthens the conclusions drawn from the SX-ARPES measurements. I therefore recommend publication of this work in Nature Communications.

.Response: We thank the reviewer for his high estimate of our work.

Reviewer #2 (Remarks to the Author):

The revised manuscript is much improved. The SX-APRES data are better explained and the new evidence in transport anisotropy of the saturation drift velocity and current further supports their interesting finding. I view this paper will not only be important for developing new strategies to improve the performance of GaN-HEMTs, but also contributes to understand interfacial 2DEGs of general interest. I recommend the publication of this manuscript for Nature communications.

Response: We thank the reviewer for such a high estimate of our work.

Reviewer #3 (Remarks to the Author):

In responding to the reviewers' comments, the revised manuscript by Lev et al. has been substantially improved. Especially the new section on transport experiment provides further support to their conclusions. I recommend acceptance of the current manuscript for publication in Nature Communications after considering some minor suggestions.

Some parts of the main text are too detail for the readers to grab the main ideas smoothly. The authors use a large paragraph (lines 60~74) to explain the advantages of the SXARPES technique, including some aspects like chemical resolution is not relevant to this study. The authors may considering shorten this part, and move the details to the methods section.

Response: To address this final point of the reviewer, we have shortened this paragraph and moved some details to Methods.

We would like again to sincerely thank all the reviewers for their highly valuable and professional work. Without their joint insist on detailed investigation of the 2DEG anisotropy effects in electron transport our work would not be really complete and have such a high appeal for practical devices.

On behalf of the co-authors,

Dr. Vladimir N. Strocov
